# Immunophenotyping myelodysplastic neoplasms: the role of flow cytometry in the molecular classification era

**DOI:** 10.3389/fonc.2024.1447001

**Published:** 2024-10-31

**Authors:** Evgenia Verigou, Theodora Chatzilygeroudi, Vasileios Lazaris, Anne-Lise de Lastic, Argiris Symeonidis

**Affiliations:** ^1^ Hematology Division, Department of Internal Medicine, General University Hospital of Patras - School of Medicine, Patras, Greece; ^2^ Division of Hematological Malignancies, Department of Oncology, Sidney Kimmel Comprehensive Cancer Center, Johns Hopkins University, Baltimore, MD, United States; ^3^ Hematology Department, General Hospital Nicosia, Strovolos, Cyprus; ^4^ Laboratory of Immunohematology, School of Medicine, University of Patras, Patras, Greece

**Keywords:** myelodysplastic syndromes, myelodysplastic neoplasms, flow cytometry, immunophenotype, molecular genetics, classification, diagnosis, prognosis

## Abstract

The unique heterogenous landscape of myelodysplastic syndromes/neoplasms (MDS) has resulted in continuous redefinition of disease sub-entities, in view of the novel translational research data that have clarified several areas of the pathogenesis and the progression of the disease. The new international classifications (WHO 2022, ICC 2022) have incorporated genomic data defining phenotypical alterations, that guide clinical management of specific patient subgroups. On the other hand, for over a decade, multiparameter flow cytometry (MFC) has proven its value as a complementary diagnostic tool for these diseases and although it has never been established as a mandatory test for the baseline evaluation of MDS patients in international guidelines, it is almost universally adopted in everyday clinical practice for the assessment of suspected cytopenias through simplified scoring systems or elaborate analytical strategies for the detection of immunophenotypical dysplastic features in every hematopoietic cell lineage in the bone marrow (BM). In this review, we explore the clinically meaningful interplay of MFC data and genetic profiles of MDS patients, to reveal the currently existing and the potential future role of each methodology for routine clinical practice, and the benefit of the patients. We reviewed the existing knowledge and recent advances in the field and discuss how an integrated approach could lead to patient re-stratification and guide personalized management.

## Introduction

Myelodysplastic syndromes (MDS) or Myelodysplastic Neoplasms are clonal hematopoietic stem cell disorders, characterized by hierarchical somatic mutations establishing ineffective hematopoiesis and by an increased tendency of evolution or transformation to Acute Myelogenous Leukemia (AML) (1–3). From the clinical and hematological point of view, MDS display substantial heterogeneity and a great spectrum of clinical manifestations, depending on the degree of ineffective hematopoiesis/BM failure, the dysregulation of the immune system, the consequences of iron overload and very rarely on potential extramedullary disease infiltration (4–8). The classification of MDS is continuously evolving, as a result of emerging information on the biology and molecular pathogenesis of these diseases, and its prognostic relevance (9–13). The application of the appropriate diagnostic approach and the identification of the patient’s prognostic profile requires the incorporation and evaluation of many clinical and biological parameters of the disease. Cytomorphology has been traditionally and still remains the cornerstone of diagnosis since the initial description of MDS, but cytogenetics and more recently molecular genetics have become an irreplaceable part of the diagnosis, classification and prognostication of these diseases and have been incorporated in several new classification and prognostication systems.

MFC is a useful tool for hematologists, supporting the diagnosis, classification, staging, follow up and the estimation and quantification of measurable clonal residual disease (MRD) in several hematological malignancies (14, 15), such as chronic lymphocytic leukemia (CLL), other mature lymphoproliferative neoplasms, particularly of B-cell origin, as well as all types of acute leukemias (AML and acute lymphoblastic leukemia: ALL) (16–24). The identification of abnormal cellular populations in the peripheral blood, BM or tissue fluids, either at diagnosis or relapse, constitutes a routine and straightforward analysis and highlights the role of MFC in everyday clinical practice for the diagnosis, prognosis and management of the afore-mentioned diseases. However, that is not the case for MDS.

Immunophenotyping dysplastic cells by MFC has become a useful diagnostic tool for MDS mainly the last 15-20 years, and although it can provide a rapid evaluation of dysplastic hematopoiesis in the BM, it has never been considered as a mandatory test for the establishment of diagnosis and/or the evaluation of prognosis for this group of diseases. The description of dyserythropoiesis or dysmyelopoieis in the BM by MFC, in a clinically meaningful way, has been the subject of investigation for more than 20 years and tremendous progress has been reported (25–28). Detection and quantification of the immunophenotypic features of dysplasia has been the endpoint of thorough investigation by several scientific groups, which, on top of reporting their findings, in several occasions have also proposed guidelines for MFC analysis or they have developed various diagnostic and prognostic immunophenotypic scores (29–32). In general, the analytical process and interpretation of the results is clearly easier for patients with excess of BM blasts, and more cumbersome for patients with low blast counts. Most recent recommendations addressing analytical issues for the evaluation of MDS were published in 2023 by European LeukemiaNet (ELN)/international MDS flow cytometry working group (iMDSFlow) (28) and are summarized in **Table 1**.

**Table 1:** ELN/international MDS flow cytometry working group 2023 recommendations for the assessment of suspected MDS bone marrow samples.

Immunophenotypic aberrancies per cellular lineage				
Markers	Blasts	Erythrocytes	Granulocytes	Monocytes
SSc	-	-	decreased	decreased
percentage	increased	increased	decreased	-
CD45	Increased CD45dim %, increased CD45-CD34+ %	-	-	-
CD34	Increased %, increased CD34bright %	-	Asynchronous expression	-
CD117	Aberrant CD34/CD117 ratio	Increased or decreased (%) of CD117+	-	-
CD123	Increased CD123+CD34+ (%)	-	-	-
CD38	Increased CD38-/dim CD34+ (%)	-	-	-
HLA DR	Increased HLADR-/dim CD34+ (%)	-	-	decreased
CD13	Increased CD13-CD33+ or CD13+CD33-	-	Aberrant pattern	decreased
CD11b	High expression on CD34+	-		Decreased
CD16	-	-		
LYMPHOID (CD2, CD7, CD5, CD4, CD56, CD19)	Decreased CD34+CD19+ (%), aberrant expression on CD34+ and/or CD117+	-	Cross lineage expression	Cross lineage expression (CD2, CD56)
CD71	-	Increased CV, decreased MFI	-	-
CD235a (optional)	-	Disturbed relationship with CD71	-	-
CD36	-	Increased CV	-	Decreased
CD105 (optional)	-	Increased or decreased (%) of CD105+ Decreased or increased MFI	-	-
CD33	-	-	Decreased MFI	-
CD10	-	-	Aberrant pattern	-
CD15	-	-		Decreased
CD14	-	-	-	Disturbed relationship with CD36
CD64	-	-	-	Decreased

Analysis requirements: 17 core antibodies, minimum number of cells: 100.000 and minimum number of CD34+ cells: 250

At the same time the substantial progress in the molecular and pathogenic characterization of the disease has allowed to better understand and to precisely classify and prognostically categorize individual patients, while offering a chance for tailored treatment (33–36). The most recent World Health Organization (WHO) and international consensus classification (ICC) classification systems recognize certain MDS entities exclusively by their molecular background as depicted in **Table 2**.

**Table 2:** Evolution of the international classification of Myelodysplastic Syndromes/Neoplasms.

FAB 1982	WHO 2001	WHO 2008	WHO 2016	WHO 2022	ICC 2022
	MDS associated with isolated del(5q)	MDS associated with isolated del(5q)	MDS with isolated del(5q)	MDS with low blasts and isolated 5q deletion (MDS-5q)	MDS with del(5q) [MDS-del(5q)]
Refractory anemia with ring sideroblasts (RARS)	Refractory anemia with ringed sideroblasts (RARS)	Refractory anemia with ring sideroblasts (RARS)	MDS with ring sideroblasts (MDS-RS) • MDS-RS with single lineage dysplasia (MDS-RS-SLD) • MDS-RS with multilineage dysplasia (MDS-RS-MLD)	MDS with low blasts and SF3B1 mutation (MDS-SF3B1)	MDS with mutated SF3B1 (MDS-SF3B1)
	Refractory cytopenia with multilineage dysplasia and ringed sideroblasts (RCMD-RS)				
				MDS with biallelic TP53 inactivation (MDS-biTP53)	MDS with mutated TP53* MDS/AML with mutated TP53*
					MDS, NOS without dysplasia
Refractory anemia (RA)	Refractory anemia (RA)	Refractory cytopenia with unilineage dysplasia (RCUD): (refractory anemia [RA]; refractory neutropenia [RN]; refractory thrombocytopenia [RT])	MDS with single lineage dysplasia	MDS with low blasts (MDS-LB)	MDS, NOS with single lineage dysplasia
	Refractory cytopenia with multilineage dysplasia (RCMD)	Refractory cytopenia with multilineage dysplasia (RCMD)	MDS with multilineage dysplasia		MDS, NOS with multilineage dysplasia
				MDS, hypoplastic (MDS-h)	
Refractory anemia with excess blasts (RAEB)			MDS with excess blasts (MDS-EB) • MDS-EB-1 • MDS-EB-2	MDS with increased blasts (MDS-IB) • MDS-IB1 • MDS-IB2 • MDS with fibrosis (MDS-f)	
	Refractory anemia with excess blasts-1 (RAEB-1)	Refractory anemia with excess blasts-1 (RAEB-1)			MDS with excess blasts (MDS-EB)
	Refractory anemia with excess blasts-2 (RAEB-2)	Refractory anemia with excess blasts-2 (RAEB-2)			MDS/AML
	Myelodysplastic syndrome, unclassified (MDS-U)	Myelodysplastic syndrome—unclassified (MDS-U)	MDS, unclassifiable (MDS-U)		
Refractory anemia with excess blasts in transformation (RAEB-T)#					
Chronic myelomonocytic leukemia (CMML)#					

*Under the category of myeloid neoplasms with mutated *TP53.*

#They are not considered MDS anymore.

Consequently, the major questions regarding the role of MFC in MDS remain: first, are there any individual cases, for which MFC analysis is necessary to establish the diagnosis? and what immunophenotypic findings are the most relevant in each scenario? Second, does MFC analysis hold any independent prognostic added value, regarding the clinical course of the patients? And lastly, can we define the clinical setting, in which MFC findings will affect drastically the management of patients?

## Aim of the review

In this review, we have explored the relationship between cellular immunophenotypic, molecular and cytogenetic features that have prognostic importance, distinct clinical presentations and outcomes in MDS patients. We have specified the disease sub-entities or sub-groups for which MFC analysis might be more useful and potentially mandatory for the confirmation of the diagnosis. Moreover, we have reviewed areas of diagnosis, treatment evaluation, and prognosis in which MFC analysis contributes to the correct decision-making and analyzed current limitations and future perspectives.

## Methodology

To achieve the above-mentioned aims of this review, a narrative PubMed/Medline review on MDS, flow cytometry and molecular findings with diagnostic or prognostic importance was performed for articles published from January 1980 until March 2024. We searched the Medline database for articles published in English using the search term ‘myelodysplastic syndromes’ adding each of the following keywords: ‘flow cytometry’, ‘immunophenotype’, ‘diagnostic score’, ‘scoring system’, ‘classification’, ‘molecular’, ‘genetics’, ‘karyotype’, ‘prognosis’, ‘diagnosis’, ‘clinical’, ‘erythroid dysplasia’, ‘erythropoiesis’, ‘monocytes’, ‘megakaryocytes’, ‘granulocytes’, ‘immature cells’, ‘peripheral blood’, ‘bone marrow’, ‘algorithm’. Articles were also obtained via cross-reference checking.

## Historical perspective

Historically, morphology had been the cornerstone of the diagnosis of MDS and the past decades cytogenetics provided new insights into the biology and clinical course of the disease, associating genotype with phenotype. Clonal cytogenetic abnormalities are detected in 40-50% of patients with primary MDS and in 80-95% of patients with secondary MDS (37, 38).

The detection of clonal chromosomal abnormality is a significant diagnostic finding proving the existence of clonal hematopoiesis. Some of the cytogenetic abnormalities, i.e. isolated deletion 5q, are related to the molecular pathogenic mechanisms of MDS, some others are secondary events, related to leukemic transformation while the significance of a small number of them has not yet been completely evaluated (10, 39).

Even though cytogenetics can confirm the diagnosis in a more robust way for the majority of patients, the need for further investigation in certain cases remains, and to this point refined MFC analysis aims to fill this diagnostic gap and/or add new information.

Early applications of Flow Cytometry analysis in the field of MDS included the demonstration or clarification of the lineage of origin of an immature cell population, not easily identifiable by cytochemistry or immunohistochemistry (40, 41), as well as the clarification of the various hematopoietic cell lineages involved in the clonal process (42). It also included the identification of missing expression of characteristic maturation antigens from the cell surface of committed or mature hematopoietic cells or the aberrant expression of such antigens on cells that should not normally be expressed (e.g. lineage infidelity) (43, 44). Flow cytometry analysis has also been used for the determination of DNA ploidy of the abnormal cells or the estimation of median DNA cell content, and for the determination of immature cell growth fraction, by testing S-phase specific biomarkers, such as BrdU, PCNA, CD71 or Ki-67 expression (45, 46). The identification of hypodiploid cells has been associated with dismal prognosis (47, 48). Additional early applications were the determination of an immunophenotypic profile for the various types of clonal cells (erythroblasts, myeloblasts, megakaryocytes), as well as of the mature cells, including erythrocytes, monocytes and platelets, the enumeration of blast cell percentage and the distinguishment of clonal hematopoietic cells from non-clonal cells infiltrating the marrow and from marrow stromal cells, mesenchymal cells and other cell types of the hematopoietic microenvironment (43, 49–53). Moreover, flow cytometry has contributed to the identification of various metabolic abnormalities of the clonal hematopoietic cells (54, 55), resulting in impaired function (54–56), apoptosis (57), oxidative stress (58), inflammatory status, senescence, mitochondrial damage, important regulatory protein expression (*TP53*, cytochrome c, hemoglobin, growth factor receptors etc) (59–62). Finally, flow cytometry has been used for immunophenotyping of peripheral blood lymphocyte subpopulations and of the bone marrow lymphoid component, as well as for the investigation of hematopoietic cell immunophenotypic alterations, following chemotherapy (particularly with alkylating or hypomethylating agents) and radiotherapy (63–65). **Table 3** resumes these initial applications of flow cytometric analysis of MDS.

**Table 3:** Early MFC applications in MDS research and clinical practice.

Early applications of Flow Cytometry analysis in the field of MDS
Discrimination of true MDS from PNH, Aplastic anemia and other diseases with bone marrow failure
Identification of the lineage of origin of an immature cell population
Determination of “abnormal” maturation and differentiation immunophenotype
Confirmation of morphological and functional abnormalities of mature cells
Determination of the normal, lineage-specific cellular immunophenotype
Detection of non-hematopoietic cells in the marrow
Demonstration of lineage involvement in the dysplastic clone
Recognition of the immunophenotype of marrow stromal cells, mesenchymal cells, fibroblasts, etc
Determination of DNA ploidy ➢ Detection of aneuploidy ➢ Estimation of S-phase cell fraction
Estimation of the S-phase fraction with specific proliferation markers (BrdU, CD71, PCNA, Ki-67 etc.)
Estimation of BM and peripheral blood blast cell percentage
Detection of proapoptotic and apoptotic cells
Quantification of the oxidative stress level through estimation of ROS
Evaluation of various aspects of cellular metabolism ➢ inflammatory reaction, mitochondrial damage, telomere length, senescence, etc.
Detection and quantification of important proteins of cellular metabolism ➢ Cell cycle regulators, Growth factor receptors, bcl2, P53, cytochrome c, ferritin etc.
Analysis of chemosensitivity against cytotoxic or non-cytotoxic agents
Evaluation of peripheral blood and BM lymphocyte subpopulations

## Immunophenotyping dysplasia

Since 2008, the WHO classification of hematological neoplasms has recognized the need of extended, secondary diagnostic criteria for the identification of clonal dysplastic hematopoiesis, characterizing MDS, to distinguish borderline MDS cases from benign, non-clonal, reactive or autoimmune conditions mimicking MDS, as well as various pre-neoplastic clinical entities such as idiopathic cytopenia of undetermined significance (ICUS), clonal cytopenia of undetermined significance (CCUS), clonal monocytosis of undetermined significance (CMUS) or idiopathic dysplasia of unknown significance (IDUS) (10, 66).

The study of antigenic expression of clonal hematopoietic cells of MDS patients by MFC has revealed abnormal antigen expression profiles in all the maturation and differentiation stages of hematopoietic cell lines, but no single antigen is considered pathognomonic to myelodysplasia (67–71). These abnormalities are: aberrant antigen expression of myeloid and erythroid cells and abnormal hyper- or hypo-expression of some specific antigens. These abnormal profiles show impressive repeatability in MDS patients and are distinct for each maturation stage (69, 72).

Technically, MFC is capable of producing large datasets from a single sample analysis, providing multi-dimensional data for every cell analyzed. Although one can easily realize why this can be a great advantage, considering the unique capability of depicting complicated patterns of cell surface antigen expression in every stage of cellular maturation in the BM, it might also become a great disadvantage. MFC requires a detailed technical surveillance of both, sample preparation and analytical protocols, and relies vastly on the expertise of the analyst. In addition, different instrumentation and reagents may produce heterogenous results and interlaboratory standardization and harmonization may be needed, in order to overcome data heterogeneity and produce comparable and reproducible results (73, 74).

In 2008, the first ELN working conference on flow cytometry in MDS took place, with the aim to standardize MFC in MDS. To that end, thirty participants from 18 institutes throughout Europe, working within the ELN and 3 experts from outside Europe (USA and Japan) joined this meeting, to define the role of MFC in diagnosis and prognostication of MDS in relation to the validated French-American-British (FAB) and WHO classifications, and to international prognostic scoring system (IPSS) and WHO classification-based prognostic scoring system (WPSS) systems; (b) to discuss the optimal methods of sample processing and handling; (c) to propose a consensual minimal set of monoclonal antibodies capable to assess dysplasia by MFC of BM cells in known or suspected MDS cases; (d) to consider the specificity of MFC analysis for MDS, related to a series of other hematologic benign or malignant diseases and (e) to suggest additional recommendations on MFC, to further optimize analysis for future directions (69). In 2012, after the WHO 2008 acknowledgement that the presence of more than 3 immunophenotypic aberrancies might be indicative of MDS, the ELN working group alongside with an international consortium cooperated to (a) define the minimal requirements to assess BM dysplasia by MFC, both, in immature progenitor cells and in the maturing myelomonocytic lineage in known or suspected MDS; (b) define how these data are to be captured, that is, how to focus on the population of interest and how they should be interpreted objectively; (c) consider the specificity of MFC analysis for MDS, as compared to a series of other either clonal or non-clonal hematological diseases, and (d) define the role of MFC in the diagnosis and prognosis of MDS in relation to the validated prognostic systems, including relevant prognostic cytogenetic and molecular markers (75).

At the same time, the Euroflow consortium (EuroFlow) evaluated the use of MFC for the detection and characterization of abnormal hematopoietic cell populations in the BM of patients with hematological malignancies and suggested an antigenic panel of 8 colors for the detailed immunophenotyping in suspected MDS and AML cases (76).

**Figure 1:**
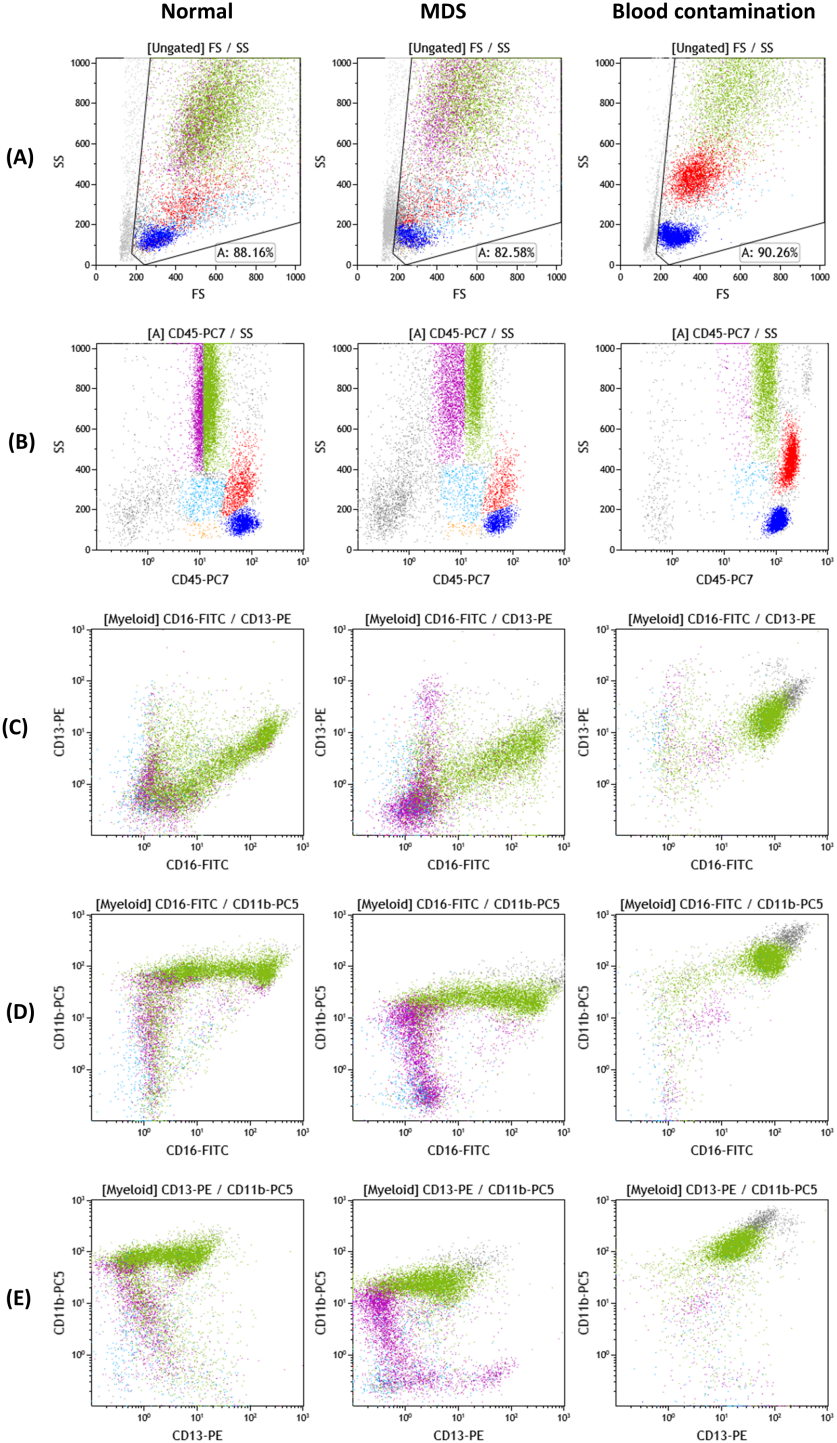
Myeloid cell antigen expression patterns of CD16/CD11b/CD13 and CD45 on normal, MDS and blood-contaminated BM samples. Color dot plots of antigenic expression of CD45, CD11b, CD16 and CD13 on bone marrow myeloid cells. Cellular populations depicted: lymphocytes (blue), monocytes (red), myeloid progenitor cell compartment (light blue), lymphoid progenitor cell compartment/hematogones (orange), maturing granulocytes (CD45dim: purple, CD45normal: green). The myeloid progenitor cell compartment and maturing granulocytic compartment constitute the “myeloid” gate. Dot plots of side scatter/front scatter [SSc/FSc] **(A)**, SSc/CD45 **(B)**, CD13/CD16 **(C)**, CD11b/CD16 **(D)** and CD11b/CD13 **(E)** from normal bone marrow, MDS and a sample with significant blood contamination exhibit three different maturation/differentiation patterns of maturing granulocytes. (normal **B–E)**: The blood contaminated sample contains only neutrophils (CD45normal to bright, CD16bright, CD11b bright, CD13normal to bright) and every other granulocytic maturation stage is absent from the analysis. The MDS sample exhibits significant variation from normal: maturing granulocytes with decreased MFI of CD11b and CD45 (normal **D**, **B** versus MDS **D**, **B)**, increased CVs of CD45, CD13, CD11b, CD16 and aberrant patterns of co-expression of the three myeloid antigens (normal **C–E**, MDS **C–E)**. Plots were obtained from Kaluza 2.1 software (Beckman Coulter; RRID: SCR_016182).

The proposed analyses constituted the basis for the implementation of multiparametric panels in clinical laboratories for the evaluation of suspected MDS from BM samples. The aim of these guidelines was to provide a common and validated strategy for the detection of marrow myeloid progenitors, the quantification of their percentage and the qualitative assessment of the different cellular populations at various stages of maturation and differentiation, as well as to compare their expression profiles with normal antigen expression patterns and with known alterations suggestive of dysplastic hematopoiesis. Most of these alterations have been thereafter, validated in several studies, providing the basis for MFC diagnostic algorithm for MDS (32, 77). Characteristic examples of antigen expression aberrancies on myeloid progenitors are: the asynchronous expression of mature and immature antigens e.g. co-expression of CD34 and CD10, the expression of infidelity markers (antigens normally expressed on other cell lineages) e.g. expression of CD7, which is a T lymphocyte marker on myeloid progenitors. For maturing cellular stages, dysplasia is commonly depicted by altered scatter characteristics, e.g. decreased side scatter in neutrophils and/or the hypo- or hyper-expression of common myeloid markers (e.g. CD16, CD13, CD11b, CD33, CD10) (68–71). Some characteristic disrupted patterns seen in dysmyelopoiesis are depicted in **Figure 1**.

## MFC diagnostic scores

These multiparametric analyses have highlighted the diagnostic utility of flow cytometry in MDS, but rely vastly on the analyst’s expertise and are time- and cost-consuming, rendering the development of flow cytometric scores the next rational step for the implementation of practical and easily-obtained diagnostic tools, that would overcome the complexity of the previous protocols (30, 31, 78–86).

The Wells algorithm (87) resulted in a flow cytometry scoring system (FCSS) that allows a simple numerical display of the myeloid and monocytic dyspoiesis in the BM that can be correlated with the IPSS and the outcome following hematopoietic stem cell transplantation. The Ogata score has been one of the most universally applied attempts to develop a clinically useful and easily implemented tool, by minimizing parameters and complexity at the same time, targeting the low grade MDS patient population without distinctive diagnostic features (without excess of blasts or ringed sideroblasts) (29, 88). Ogata score utilizes a minimal MFC panel of four parameters, created from one cell sample stained with two fluorescent antibodies, anti-CD34 and anti-CD45 (29, 85). It is technically simple and reproducible by other groups (83, 89–92), but its sensitivity and specificity in diagnosing low grade MDS is limited. Thus, the authors later proposed a new parameter: the CD33 expression on CD34+ myeloid progenitors and maturing granulocytes (86) which has showed 50% sensitivity and more than 95% specificity in low grade MDS. Our group has proposed a dysmyelopoiesis flow score (DMI) that has 77% sensitivity and 93.6% specificity for low grade MDS, indicating the presence of multilineage dysplasia in certain cases, which were falsely characterized as refractory anemias by cytomorphology evaluation (30).

Erythroid cells exhibit commonly some of the most characteristic dysplastic morphological features in MDS and that is why many groups have tried to incorporate immunophenotypic alterations seen in dyserythropoiesis in flow scores, to increase the sensitivity of low grade MDS detection (31, 82–84, 93, 94).

## Immunophenotypic findings in MDS

The detailed description of immunophenotypic aberrancies on the surface or the cytoplasm of cells of different cellular lineages in MDS has been the basis for the development of every diagnostic tool or algorithm, proposed up to date. The majority of the proposed indices of dysplasia have used BM samples since the abnormal expression patterns are recognized, not only on mature and terminally differentiated cells, but also on the immature marrow progenitors.

### Myeloid progenitors

One of the most common requests in flow cytometric analysis of confirmed or suspected MDS is blast cell quantification. Estimation of blast cell percentage by morphology discriminates MDS from AML and further defines prognostic relevant subcategories, according to both, WHO classification and revised international prognostic scoring system (IPSS-R). MFC can quantify and characterize immature cells, but it is important to note that their percentage although correlates, it is not identical with the morphological findings (95). Visually characterized myeloid blasts do not always correspond to immature cells in MFC, that are traditionally defined as CD45weakCD34+CD117+ HLADR+ (96, 97). This discrepancy can stem from the removal of erythrocytes from the phenotypic analysis but is mainly attributed to the fact that morphologically identified blasts can be more mature and differentiated cells than myeloblasts, sometimes referred to, as blast equivalents, such as neoplastic promonocytes or promyelocytes in certain cases of acute leukemias, a term adopted by the ICC in the latest classification (12). Another confounding factor can be hemodilution of the sample that leads to artificially lower blast percentages. Nonetheless, a multicenter study of the ELN iMDSFlow WG, published in 2023, confirmed that a percentage of ≥3% marrow myeloid progenitors (CD45weak/SSClow/CD34+/CD19) strongly suggests the existence of MDS or myelodysplastic/myeloproliferative neoplasms (MDS/MPN) (77).

The added value of MFC lies in the ability to characterize the abnormal cell compartment for aberrant expression of antigens that can be infidelity markers (CD2 and CD7 expression on myeloid cells), asynchronously expressed, overexpressed or lacking (e.g. lack of CD34 expression on CD117+ cells or overexpression of CD117, which is associated with worse survival) (98). These findings can define myelodysplasia irrespective of the actual blast cell percentage.

### Myeloid and monocytic lineage

The combination of side scatter (SSC) and CD45 expression constitutes the main gating strategy for the myeloid populations that are affected, along with the erythroid lineage in the context of MDS. In some cases, in which these populations are hard to be defined, auxiliary markers, such as CD64 or CD33 can be utilized.

The low SSC exhibited by granulocytes is well-described and correlates with morphological dysplastic features, such as hypogranulation. It is one of the main parameters of the Ogata score, suggesting that the ratio of granulocyte SSC to lymphocyte SSC offers a more sensitive and quantifiable marker of dysplasia (85, 86). Since this is a simple and not reagent-demanding analysis, it can be implemented to the study of any sample suspicious for MDS before any further analysis. Moreover, the Ogata score is recommended by the iMDSFlow as a preliminary assessment for MDS and this has been validated by several publications (89, 91, 99, 100).

In terms of fluorescence, the co-expression patterns of CD13/CD11b and CD16/CD11b can offer very important information, concerning abnormal maturation and differentiation of the myeloid compartment, as they appear, are co-expressed and disappear creating specific patterns in normal BM (30, 76). These patterns are reliable indices of dysplasia, provided that the sample quality is acceptable. Some characteristic disrupted patterns seen in dysmyelopoiesis are depicted in **Figure 1**, compared to normal findings and a blood contaminated BM sample. Additional insight is offered by the cytoplasmic markers MPO (myeloperoxidase- found in primary granules) and LF (lactoferrin- secondary granules), along with CD10 and CD15, that define mature granulocytes (101). Severely altered expression of MPO/LF has been correlated with low-risk MDS and can distinguish them from normal samples (102). It is also important to delineate the potential aberrant expression of infidelity markers, such as CD56 or other lymphoid indices in dysplastic granulocytes (77). From recent reports, the most sensitive marker for granulocytes, with a specificity of nearly 100% in the dysplastic granulocytic marrow compartment, is an abnormal SSC distribution, whereas abnormal maturation profiles through CD13/CD11b, CD16/CD11b and CD16/CD13 co-expression patterns, along with aberrant expression of CD2 CD7, CD5 and CD19 occur in about 70% of the cases (69, 75).

The monocytic lineage presents a variety of phenotypic disorders. The typically expressed markers CD36, CD14, CD16, CD11b, HLADR, CD13, CD33 can be lacking or have an abnormal expression (77, 89, 103–105).

The aberrant overexpression of CD56 is also frequently observed, although it can be also found in reactive/regenerative conditions of the BM (89). CD14 and CD16 expression defines three distinct monocyte sub-populations (classical monocytes CD14+CD16-, intermediate CD14+CD16+ and atypical CD14weakCD16+), whose relative percentages can be disturbed in dysplasia, chronic myelomonocytic leukemia (CMML) and in reactive/inflammatory conditions. This distribution is the most sensitive marker in CMML and is more pronounced in the peripheral blood, where a cutoff >94% of classical monocytes has been proposed to strongly point to CMML (103).

### Erythroid lineage

The erythroid lineage, delineated by negative CD45 and low SSC, is another target for flow cytometric analysis in the context of MDS. Anemia and transfusion dependency remain one of the most frequent presenting features triggering the MDS diagnostic investigations. In classical MFC protocols, RBC lysis preceding analysis, shortens the erythroid compartment that remains, which, along with the paucity of available markers, renders the analysis suboptimal (84). Therefore, efforts have been made to develop non-lysis protocols, that preserve all erythroid cells (83, 94) and several new markers have recently been tested. The initially reported hypoexpression of CD71 and CD235a have been supplemented with the study of CD36, CD117 and CD105, providing new information on the development of the erythroid lineage. In 2013, Mathis et al. developed the RED score for dysplasia, using hemoglobin levels, CD71 and CD36 expression (83), which, when combined with the hepcidin:ferritin ratio appears to predict response to erythropoietin (EPO) in lower-risk MDS (82). In 2017 the iMDS Flow Group reported that an increased coefficient of variation (CV) of CD36 and CD71 constitute the best indicators of dyserythropoiesis (84) and Cremers and al. incorporated their findings to the existing diagnostic MFC scores, to improve their sensitivity (31). In 2019, Violidaki et al. developed a protocol using CD36, CD71, CD105, CD117, CD13 and CD45, combined with a no-lysis protocol, which provided a more reliable evaluation of erythroid dysplasia and led to the integration of artificial intelligence (AI) in the form of Flow-Self Organizing Maps algorithm (FlowSOM) unsupervised analysis, to define distinct clusters within the erythroid compartment in normal BM and subsequently in MDS (94, 106, 107). This analysis demonstrated that the abnormal clones gradually decreased after treatment with azacytidine and were nearly normalized after allogeneic hematopoietic stem cell transplantation (HSCT).

### Megakaryocytes and platelets

Megakaryocytes, mainly due to their size, fragility and scarcity in the BM aspirates, have not been extensively studied in the context of MDS. Focusing mainly on megakaryoblastic leukemias, the EuroFLOW has included the megakaryocytic/platelet markers CD61, CD41 and CD42a in the AML/MDS panel, albeit not considering them beneficial for the evaluation of suspected MDS (76).

CD41 (or Glycoprotein IIb-GPIIb) has been evaluated in the context of MDS blasts. It may be present at diagnosis or appear during disease progression, has been detected in several cases with unfavorable prognosis and appears to be associated with monosomies or complex karyotype (108, 109).

Due to the above-mentioned difficulty of megakaryocyte analysis, efforts have been made to evaluate peripheral blood platelets as potential indicators of dysplastic thrombopoiesis. From the markers evaluated in a study with 83 participants (44 MDS, 20 healthy subjects and 19 patients with non-MDS conditions), CD61, CD36 and CD42a were found decreased and CD34 was asynchronously expressed within the MDS group (110). Although the authors have proposed a diagnostic score, these observations have not been further validated and widely accepted.

### Lymphoid compartment

An often-mentioned phenotypic characteristic of the lymphoid BM subpopulations in MDS is the relative decrease of lymphoid progenitors (69). It is included in the Ogata score (as <5% B-progenitors in all CD34+ cells) (59), but has been excluded when the score was revised (86).

The immunological alterations of the dysplastic BM are plenty and can give rise to both, a pro-inflammatory and an immunosuppressive profile (in lower- and higher-risk MDS, respectively (111, 112). This in turn is expressed by altered relative percentages of T cell sub-populations, along with cells of the dendritic cell compartment. CD8+ cytotoxic T-lymphocytes are found increased in the peripheral blood and BM of lower-risk myelodysplastic syndromes, reflecting an anti-tumor attempt that ends up by suppressing both, the malignant and benign hemopoiesis (113–115). In this instance, Th17 cells are increased and Tregs are low, whereas in more advanced stages of the disease this profile shifts to a more immunosuppressive one, with high numbers of Tregs, and Th22 lymphocytes, and low CD8+ T cells, and with immune checkpoint molecules upregulation (116, 117).

### Proliferation and apoptosis

The use of markers detecting apoptosis or cell proliferation has long been used as a standard practice in the histopathologic examination of hematologic malignancies but has been less established with MFC. Several types of markers and MFC protocols have been evaluated for the evaluation of proliferation (118, 119) and apoptosis (120–122) but, in contrast to other neoplastic diseases, the pathways and the biology of cell survival and death in MDS are variable and multifactorial. Many studies have demonstrated that in the early stages there is a high apoptotic index, that later shifts to a high proliferation/low apoptosis state, as the leukemic clone is progressing, and this is also correlated with IPSS (123–125). Pathways of pyroptosis, detected cytometrically by the expression of casp-1, appear to participate in the early apoptotic stages (126, 127) possibly through toll-like receptor 4 or CD33. In a review article by Menstrum et al., 18 studies (each of them analyzing >10 subjects) have been assessed for the prognostic potential of proliferation/apoptosis analysis. The results have confirmed the afore-mentioned observations and have detected correlations with OS, PFS and response to treatment for MDS and AML (128).

The recent standardization of MFC by EuroFlow could allow the integration of such diagnostic markers of cytometric evaluation in the clinical setting. Nies et al. proposed an MFC assay to evaluate proliferation rate, during maturation of various hematopoietic lineages in the normal BM (129) and attempts have been made to implement this assay as a diagnostic tool (130).

## Differential diagnosis and prognostic impact

There are several clinical entities sharing overlapping clinical and/or laboratory features with those of MDS, raising a, sometimes urgent, diagnostic problem. Cytopenias and especially pancytopenia may accompany various infections or other inflammatory conditions, in association with morphologically atypical cells. In these cases, MFC may offer a solution to the diagnostic problem, restricting the need for more advanced, expensive and resource-consuming investigations, by the enumeration and immunophenotypic characterization of myeloid progenitors and the delineation of normal/abnormal or aberrant maturation patterns.

In the case of paroxysmal nocturnal hemoglobinuria (PNH) that can accompany other cytopenia-inducing conditions, including MDS, MFC remains the gold standard for diagnosis, by confirming the loss of the glycosylphosphatidylinositol (GPI)-anchored proteins CD55 or CD59 on the surface of red cells or CD16 and fluorescein-labeled proaerolysin (FLAER) on granulocytes, within hours (131).

Aplastic anemia represents a challenge in morphological evaluation, due to the severe hypoplasia of all BM cellular compartments. Although MDS usually presents with hypercellular or normocellular BM, in 10-15% of cases the biopsy detects hypocellularity (132). Nonetheless, due to the ability of MFC to detect and evaluate large numbers of cells, an accurate percentage of progenitors can be deduced, as well as dysplastic features in the granulocytic and erythroid lineages. Moreover, it is a relatively easy evaluation, which can be repeated whenever any potential disease progression is suspected.

In the context of CMML and pre-CMML conditions, apart from a first evaluation of the BM blast percentage, aiming to exclude AML, and the evaluation of monocyte subsets, as was described earlier, neoplastic monocytes exhibit frequently an aberrant expression of CD56, CD2, CD5, CD10, CD23 or under-express CD13, CD14, CD15, CD33, CD38, CD45, and CD64 (133). Bearing in mind that phenotypically abnormal monocytes may also occur in other myeloid neoplasms, they are nonetheless typically neoplastic cells and warrant further investigation. A special mention should also be made on systemic mastocytosis associated with CMML, in which mast cells invariably express CD25 on flow cytometric analysis (134).

Perhaps the most abundantly discussed problem, concerning MDS, is their differential diagnosis with ICUS and CCUS. Although these entities may indeed represent early stages of MDS, they do not fulfill the morphological criteria of dysplasia, highlighting the ability of MFC to detect -and also monitor by serial testing- abnormal maturation patterns and clone establishment or instead to exclude MDS (135). The usually applied scores (e.g. Ogata or Wells) do not perform well in the differentiation of ICUS vs CCUS but show promise in selecting patient populations with a higher risk of progression (136). When morphological criteria for MDS were not met but MFC features indicated MDS, there was a rate of 50% of progression to overt MDS (137, 138).

For more than 15 years, MFC findings have also been tested as a prognostic tool, either by being correlated with the validated prognostic scoring systems for MDS patients or by assessing their independent prognostic value for overall survival (OS), leukemia free survival (LFS) or post-transplantation outcome in several studies (99, 138–143). These studies highlight the prognostic significance of MFC findings, incorporated in various immunophenotypical scores or sums of abnormal findings instead of single parameters tested (98, 144, 145) but until now immunophenotype is not an established tool for monitoring, staging, and predicting disease and treatment outcomes in international guidelines for MDS (146). Relatively early it has been recognized that a higher expression of HLA-DR in association with low expression of CD11b on total BN cells may predict earlier transformation to AML (147). In other instances, MFC scores have been developed that hold independent prognostic value but do not correlate significantly with IPSS–R, identifying patients with different OS, as reported by Vido-Marques et al (78). Gardikas et al. have reported that a higher rate of apoptosis in the CD34+/CD117+ myeloid compartment is an independent favorable prognostic factor for both, OS and transformation to leukemia (148). Recently, Guarnera et al. have shown that Ogata score ≥2 is significantly associated with the detection of ≥2 mutations, as well as with inferior event-free-survival in MDS patients (149). In a cohort of cytopenic patients (either with MDS or nonclonal cytopenia), without considering cytomorphology diagnosis, iFS (integrated Flow Score) and Ogata score have shown prognostic significance for overall survival. Furthermore, in cytomorphologically characterized MDS patients, multivariable Cox regression analysis including iFS, IPSS-R (as a whole or with its single components) and age has shown that iFS could add independent prognostic information, beyond IPSS-R (32). Oelschlaegel et al. recently published a new MFC strategy for screening MDS, which could be used as a treatment monitoring tool after further validation of its value (150).Future validation of these scores as well as further integration with genetic and molecular data, in tools such as the molecular IPSS would probably clarify the potential prognostic value of immunophenotype in the molecular classification era.

## Molecular classification of MDS

The first classification introducing cytogenetics features was the 3^rd^ classification of myeloid neoplasms by WHO in 2001. In this classification, MDS with isolated deletion of the long arm of chromosome 5 [del(5)q] was identified as a separate entity. Since then, the WHO’s revisions published in 2008 and 2016 have refined additional clinical entities. The introduction of *SF3B1* gene mutation in the criteria of diagnosis of MDS with ring sideroblasts and the identification of Clonal Hematopoiesis of Indeterminate Potential (CHIP), an entity without clear MDS features but with gene mutations commonly seen in MDS in the 2016 revision, reflected the great importance of the molecular characteristics (11). The latest WHO edition (2022) incorporated subtypes, based on their molecular characteristics, such as the MDS with biallelic *TP53* inactivation (MDS-bi*TP53*), or MDS with low blasts and SF3B1 mutation (MDS-*SF3B1*), which replaced the previous entity “refractory anemia with ring sideroblasts”, and finally the Clonal Cytopenia of Undetermined Significance (CCUS), which is defined as CHIP in the presence of one or more persistent cytopenias that are otherwise unexplained (13). The same year, the ICC group also suggested a similar classification while redefining the percentage of blast cell boundaries for the characterization of MDS versus AML (12). The continuous accumulation of genetic data in the classification systems in the last decade was resulted from the extended use of Next Generation Sequencing (NGS) (151). These data have been associated with morphological features and, in some cases, with specific immunophenotype alterations (152, 153).

The genetic landscape of MDS is quite complex. Several mutations have been identified in genes that are involved in RNA splicing (*SF3B1, U2AF1, SRSF2*, and *ZRSR2*), DNA methylation (*DNMT3A, TET2, IDH1/IDH2*), chromatin modification (*EZH2, ASXL1, KDM6A*), transcription (*RUNX1, BCOR, ETV6, GATA2*), cohesion complex (*STAG2*), signal transduction *(JAK2, KRAS/NRAS, CBL*), and tumor suppression (*TP53, WT1*) (33, 154). Some mutations, such as *TP53^multihit^, FLT3, MLL^PTD^, ASXL1, BCOR, EZH2, NRAS, RUNX1, STAG2*, and *U2AF1* show unfavorable outcomes regarding OS, LFS, and AML transformation. Although *SF3B1* mutations seem to have favorable outcomes, this is not always the case, when additional mutations coexist (34).

The translation of this knowledge to clinical practice was the incentive to develop the new classification systems, in an effort to more accurately describe subgroups of patients with different biological behavior, clinical presentation or prognosis and individualize our treatment strategies (155). MDS with low blast percentage and *SF3B1* mutation (MDS-*SF3B1*) is a distinct 2022 WHO and ICC classification sub-category, replacing the MDS-RS of the previous WHO revision. The patients usually have a relatively good prognosis, anemia, and a high degree of ineffective erythropoiesis (156). *SF3B1* gene encodes a protein that plays a critical role in the spliceosome machinery. Mutant *SF3B1* induces errors in the splicing process of mRNA of the genes involved in iron homeostasis, such as the *PPOX* and *ABCB7* genes. This leads to iron accumulation in the mitochondria of the erythroid progenitor cells, resulting in the formation of ring sideroblasts, the morphologic hallmark of sideroblastic anemia. In MDS carrying mutations of the *SF3B1* gene, there is also increased erythroid apoptosis due to impaired *GATA-1* expression and end-stage erythroid maturation arrest attributed to *EIF2AK1* activation (157, 158). The available therapies try to reduce erythoblastic apoptosis and alleviate anemia and its symptoms.

*TP53* mutations are present in many types of cancer. The occurrence of these mutations in MDS and AML constitutes a criterion to define separate disease categories according to the new classification systems. Thus, in the 2022 WHO classification, MDS with biallelic *TP53* inactivation (MDS-bi*TP53*), which means the presence of two or more TP53 mutations or of one mutation, with evidence of *TP53* copy number loss or copy neutral loss of heterozygosity (LOH), when BM or peripheral blood blasts are less than 20% constitutes a new clinical entity. In the ICC classification, the same entity is defined as the myeloid neoplasm with two distinct *TP53* mutations (MDS-*TP53*, MDS/AML-*TP53*), each with a variant allele frequency (VAF) of >10% or a single *TP53* mutation with one of the three following criteria (1): 17p deletion on classical cytogenetics; or (2) VAF of >50%; or (3) copy-neutral LOH at the 17p *TP53* locus (33). The MDS with *TP53* mutations is characterized by poor prognosis and chemotherapy resistance. It should be noted that patients with a single *TP53* mutation have the same prognosis as the wild type (159).

As mentioned above about 50% of patients with MDS have an abnormal karyotype. These abnormalities are mainly consisting of chromosomal deletions, monosomies, trisomies, and more rarely inversions or translocations. One of the reasonably common and well-studied cytogenetic alterations is the del(5)q MDS with del(5q) represent a unique category and was the first genomic aberration included in the WHO classification. Patients without an excess of blasts, exhibiting this abnormality alone or accompanied by an additional one, other than monosomy 7 or del(7q) tend to have several common clinical and morphologic features, steady clinical course and a quite good prognosis (160). Patients usually have macrocytic anemia, without other cytopenias or sometimes exhibit mild to moderate thrombocytosis. Their megakaryocytes have smaller size and have hypolobulated or non-lobulated nuclei. Patients respond favorably to treatment with lenalidomide, possibly due to the haploinsufficient casein kinase-1-alpha-1 (*CSNK1A1*) gene (161). This gene has a pathogenetic role in the clonal expansion of malignant cells and the disease represents an acquired ribosomopathy, resulting from haploinsufficiency of the crucial Ribosomal Protein of the Small subunit-14 (*RPS14*) gene, which resides in the deleted 5q region. Haploinsufficiency of the *RPS14* affects ribosomal assembly, impairs erythroblastic protein synthesis and results in early cell death and dyserythropoeisis. There are many more genes in the deleted chromosomal locus that contribute to the clinical and laboratory features of the disease. For instance, the haploinsufficient gene coding *miR145* through the consequent upregulation of Friend leukemia virus integration 1 (*Fli1*) gene enables the effective megakaryopoiesis and results in thrombocytosis (162).

## Association of immunophenotyping with cytogenetics and genomics

### Studies exploring the correlation between MFC findings and genetic mutations in MDS and its clinical impact

The interplay of immunophenotypical findings and genetic alterations partly reveals the association between genotype and phenotype and has been a major field of investigations for the past few years. Some studies have demonstrated an association between genotypic abnormalities and phenotypic patterns in patients with unexplained cytopenia (163). The main findings of these studies are summarized in **Table 4**. All patients with more than two cytogenetic abnormalities, monosomy 7/del(7q) or del(5q) were shown to have a positive FCSS (163). Guarnera et al. examined 106 patients with MDS and 39 controls, and using the clustering proposed by the EuroMDS score, recently reported that certain immunophenotypic aberrancies are associated with different clusters (149, 155). Ogata score ≥2 in MDS patients was found to correlate with the existence of more than 2 mutations in the molecular testing, as well as with epigenetic modifier gene mutations, such as *SRSF2* and *TET2* (149). On the other hand, Euro-MDS group 0 (without specific genomic profiles) is associated with Ogata score<2. Regarding specific markers, CD56 expression has been associated with *DMNT3A* and AML-like mutations (*NPM1, FLT3, IDH1*, and *RUNX1* genes) (Euro-MDS group 7), and CD15 expression with *U2AF1* mutations (Euro-MDS group 4). CD38 expression has been specifically associated with *TP53* mutations, whereas CD117 expression correlates with Euro-MDS group 2 (*TP53* mutations or complex karyotype) (149, 155). Moreover, decreased CD177 positive neutrophils have been associated with specific gene mutations in myeloid neoplasms, such as *FLT3-ITD*, *NPM1, NRAS, RUNX1, TET2* and *U2AF1 S34F* (135). However, this association has not been confirmed in a separate MDS patient cohort (164).

**Table 4:** Association of specific genomic alterations with characteristic immunophenotypic findings in MDS patients.

Study	Genomic alterations	Immunophenotypic findings
Cutler et al., 2011 (134)	• >2 karyotypic alterations • -7/del(7q) • del(5q)	• Positive FCSS
Guarnera et al., 2023 (135)	• >2 molecular mutations or epigenetic gene mutations (*SRSF2*) • Without specific genetic alterations • *U2AF1* • *DMNT3A* or AML-related genes • *TP53*	• Ogata score≥2 • Ogata score<2 • CD15 expression • CD56 expression • CD38, CD117 expression
Duets et al., 2021 (138)	• *SF3B1*	• Increased erythroid progenitors and mast cells • Erythroid progenitors: Decreased CD71 with increased CV, increased SSc • Neutrophils: increased CD11b • Monocytes: increased CD11b, decreased CD36, CD64
Weiß et al., 2023 (123)	• *SRSF2*	• Granulocytes: aberrant CD11b/CD16 pattern • Myeloid progenitors: decreased CD45
Oelschlaegel et al., 2015 (124)	• del(5q)	• Myeloid progenitors: increased %, low CD45 MFI ratio • Granulocytes: decreased SSc, normal CD71, CD10 • Erythrocytes: increased CD71dim (%)
Dutta et al., 2022 (143)	• *TP53*	• Blasts: increased % CD7+
Dannheim et al., 2018 (144)	• *TP53* and/or complex karyotype	• Myeloid cells: T cell antigen expression (CD3, CD5, CD7)
Chen et al., 2019 (142)	• Monosomy 7 or del(7q)	• Granulocytes: increased CD14, CD64, CD13 • Blasts: CD5, CD7, CD56 expression

Recently, the combination of fluorescence *in situ* hybridization (FISH) and flow cytometry cell sorting has identified distinct distributions of cytogenetic abnormalities across different myeloid maturation stages. Patients were divided into three categories: Patients with accumulation of alterations on immature myeloid cells, such as those with monosomy 7, patients with a single cytogenetic abnormality, detected throughout all maturation stages, such as those with good-to-intermediate prognosis (according to IPSS-R) and patients with evidence of clonal evolution. In the latter group, abnormalities representing the founding clone were evenly distributed across stages of myeloid maturation, whereas subclonal abnormalities were mostly encountered in the immature myeloid populations (165).

Various recent reports attempt to describe specific immunophenotypic profiles of key molecular or cytogenetic findings in MDS. The major findings of these studies are summarized in **Table 4**.

### Abnormalities associated with del(5q)

One of the hallmarks of MDS del(5q) immunophenotype is the increased number of myeloid progenitor cells (153). The CD45-MFI-ratio, defined as the intensity of CD45 in reference to the normal lymphocytes (87), is associated with maturation within the progenitor cell compartment and has been found to be normal-to-low in del(5q) MDS patients (153). Even though an abnormally low granulocyte SSC-ratio is observed in almost all del(5q) patients, the low degree of dysgranulopoiesis observed cytomorphologically is consistent with normal granulocyte CD71 and CD10 expression. Moreover, although previous reports have reported CD14 overexpression on the BM granulocytes (166), more recent reports did not confirm this finding (167). Lastly, CD71dim nucleated erythroid cells are distinctly higher in del(5q), compared to normal karyotype MDS patients (153).

Based on these observations, Oelschlaegel et al. proposed the 5-parameter-del(5q)-score, including CD45-MdFI-ratio (lymphocytes vs myeloid precursors; ≤7.0, 10 points), percentage of myeloid precursors (>2.0%, 3 points), granulocytes versus lymphocytes SSC ratio (<6.0, 2 points), CD71 expression on granulocytes (≤20%, 1.5 points), and sex (female, 1.5 points). A score of 15.0 or more can predict 95% of MDS harboring this abnormality whereas a score of less than 10 was not identified in del(5q) MDS (153).

### Immunophenotypic features of myeloid malignancies with chromosome 7 abnormalities

Recent data have shown that myeloid neoplasms with monosomy 7 typically demonstrate multiple immunophenotypic abnormalities on myeloid blasts and maturing myelomonocytic cells (167). In general, immunophenotypic aberrancies are more common in patients with monosomy 7, than in patients with del(7q). Specifically, increased CD14 expression on maturing granulocytic cells is the most frequent aberration associated with monosomy 7, but not with deletion 7q. Increased CD14 is observed in patients with monosomy 7 as a single abnormality, as well as in those with additional cytogenetic abnormalities. In some patients with monosomy 7, this increase in CD14 expression is present uniformly at all stages of maturation, while other patients exhibit variability of CD14 expression at different compartments of granulocytic maturation, with some of them expressing normal levels of CD14 and other demonstrating increased CD14 expression. Chen et al. hypothesized that these patterns may reflect the size of the clones harboring the chromosome abnormality. Granulocyte CD64 retention was also more frequently observed in patients with monosomy 7, than in those with deletion 7q. On the other hand, in both of these chromosome 7 abnormalities, CD13 expression is usually found increased on the granulocytic precursors and on maturing granulocytes in the majority of cases. The same finding has also been observed on granulocytes following G-CSF stimulation.

Regarding myeloid blasts, aberrant CD5, CD7 and CD56 expression are observed with substantial degree of variability in both, chromosome 7 abnormalities, alone or associated with other abnormalities (167).

### Immunophenotypic specificity of SF3B1 mutated MDS

As mentioned above, MDS -*SF3B1* is now classified as a distinct subtype, due to its favorable course and responsiveness to treatment with luspatercept (156). Favorable prognosis is further underlined by the low incidence of high (>2%) CD34+ myeloid progenitors in this MDS subtype. Moreover, Duetz et al. reported that compared to other subtypes, *SF3B1*-mutant MDS presents significantly higher percentages of erythroid progenitors and mast cells. Erythroid progenitors express less CD71 than other types of MDS, indicating that the CD71-shedding process is severely disturbed. CD71 presents high CV, that is negatively correlated to hemoglobin levels in these patients. High prevalence of ring sideroblasts is accompanied by higher erythrocyte progenitor sideward-light-scatter (SSC). Higher SSC has also been observed in myeloid progenitors and higher expression of CD11b has been reported in neutrophils. Monocytes in *SF3B1*-mutant MDS also have higher CD11b expression, along with lower expression of CD36 and CD64. Duetz et al. have clarified that this increased CD11b expression in neutrophils and monocytes, as well as higher CD36 expression and higher numbers of mast cells, are more prominent among patients with the *K700E SF3B1* mutation. Lastly, in patients with both, *SF3B1* mutation and del(5q), erythroid progenitor cell count, CD71 marker CV and mean fluorescent intensity (MFI) resembled more the del(5q) phenotype, suggesting that patients with both mutations might benefit more from lenalidomide than luspatercept (168).

### Distinct granulocyte immunophenotypic patterns of SRSF2 mutated MDS

Another gene of the splicing machinery that is of major importance in MDS is the serine and arginine-rich splicing factor 2 (*SRSF2*). Mutations of *SRSF2* are identified in 15-20% and have been associated with increased age and favorable prognosis (169, 170). In SRSF2 mutant MDS, a distinct immunophenotype was identified by Weiß et al., exhibiting a specific CD11b/CD16 co-expression pattern in granulocytes and reduced CD45 expression in myeloid progenitors. Normal granulocytic maturation initiates with an abrupt increase in CD11b expression and a parallel slight increase of CD16 expression. Subsequently, follows a steep increase in CD16 expression, paralleled by only a minimum further rise of CD11b expression (giraffe pattern). Diversely, *SRSF2* mutant MDS is characterized by an increase of CD11b without CD16 expression, followed by an increase in CD16 expression without any further increase of CD11b expression (rectangle pattern) and only a dim CD45 expression in myeloid progenitor cells (152).

### Immunophenotypic profile of TP53 mutated AML and MDS patients

Immunophenotypic profiles of *TP53* mutated MDS and AML patients are very similar, and the only difference is the higher percentage of CD7 expressing blasts, observed in MDS patients (171). Additionally, overall CD38 expression is specifically associated with *TP53* mutation and overall CD117 expression correlates with *TP53* mutation or with complex karyotype (149). Compared to normal karyotype, *TP53* mutated AML blasts exhibit increased expression of CD34, CD13 and CD5, but lower expression of CD10. Moreover, monocytes in complex karyotype or/and *TP53* mutated AML patients usually show markedly brighter expression of CD7, CD11b and CD13, as well as aberrant expression of CD34. The granulocytes of these patients demonstrate modestly higher expression of CD3, CD5, CD7 and CD14. Considering the above, aberrant expression of T-cell antigens within the myeloid lineage is characteristic of complex karyotype or/and *TP53* mutated MDS/AML (172).

### Immunophenotype of NPM1 mutated MDS/AML

Nucleophosmin (*NPM1*) mutations are present in about 30% of adult AML patients but are also present in ~4% of MDS patients (173, 174). Current classifications differ in the characterization of these patients, since *NPM1* mutation constitutes an adequate criterion for AML diagnosis in WHO 2022 classification, while the ICC requires the 10% blast percentage as a co-criterion for the establishment of AML diagnosis, thus labeling as MDS patients with a lower blast- (or blast equivalent) cell percentage. Immunophenotypic analysis of leukemic myeloid blasts in AML and MDS with excess of blasts (MDS-EB), exhibited a common pattern of leukemia associated immunophenotype (LAIP), which was characterized by CD117+ blasts with low side scatter and decreased or complete absence of CD13, CD34 and/or HLA-DR expression, increased CD33 and/or CD123 expression, as well as absence of CD15 and CD64 expression. This pattern resembles that of normal promyelocytes (CD34-, CD117+, CD33+, HLA-DR-, CD13++, CD15+, and CD64+) and of immature monocytes (CD34-, CD117-, CD33++, HLA-DR++, CD13-, CD15+, CD64+, and higher CD45), but these cells exhibit almost invariably absence of CD15 and CD64 and decreased expression of CD13 or HLA-DR. In a subset of patients leukemic blasts express the lymphoid markers CD7, CD56 or dim CD19, but are negative for CD5 (174).

In 52% of *NPM1* mutated MDS/AML an immature monocytic population was detected, which was positive for HLA-DR, variably positive for CD4, CD15, brightly positive for CD33 and CD64, and negative for CD13, CD14, CD16, CD34, or CD117 (174).

Analysis of the immunophenotype of *IDH1/2*-mutated AML/MDS-EB cases showed a highly significant association between the simultaneous presence of *IDH1/2* and *NPM1* mutations and confirmed the double negative ‘acute promyelocytic leukemia-like’ myeloid phenotype described above: lacking both, CD34 and HLA-DR and highly expressing MPO and CD33 (175).

### Immunophenotypic alterations induced by hypomethylating agent treatment and potential clinical significance

In one study improvement of immunophenotypic features was shown in 41% of MDS patients, treated with azacytidine (AZA) and in 68% of them this was accompanied by a hematological response (176). Immunophenotypic improvement was defined as one of the following: a) normalization or reduction of the number of immunophenotypic abnormalities observed on the myeloid CD34+ population, b) reduction by at least 50% of the percentage of CD34+ myeloid cells pre-AZA and normalization of the granulocytic or monocytic pattern of maturation, c) normalization of the granulocytic and monocytic patterns of maturation (without improvement of the immunophenotype of CD34+ myeloid cells), d) improvement of one maturation pattern (granulocytic or monocytic) and improvement of two of the following: erythroid cells abnormalities, normalization of monocyte percentage or correction of reduced granulation of granulocytes (combination of CD45 and SSC).

This improvement in MFC findings was associated with better clinical response after 6 cycles of AZA treatment and the probability of failure or resistance to AZA was much higher among patients not exhibiting any of these findings. Moreover, improved immunophenotypic features following AZA treatment were found to predict longer duration of hematological response in MDS patients (176).

### Potential for personalized medicine approaches, based on integrated immunophenotypic and genomic data

Integration of genomic and immunophenotypic data is still at an early stage, however the potential for personalized approaches is arising. Genetic alterations with a known clinical impact have transformed clinical practice in certain scenarios. Lenalidomide response rates in lower risk MDS patients with del(5q) are 60-65% and transfusion independence is achieved with a median duration of 2-2.5 years. On the contrary, in the same patient population anemia has shown substantially lower response rates and significantly shorter responses to erythropoietin stimulating agents (ESA), compared with other categories of lower-risk MDS (177). The 5-parameter-del(5q)-score proposed by Oelschlaegel et al. can be used to faster identify these patients before their cytogenetic confirmation, with a predictability of 95% and accelerate appropriate treatment selection (153). MDS-*SF3B1* also constitutes a distinct subtype due to its favorable course and responsiveness to luspatercept (156). This entity shows specific immunophenotypic features (138) that could contribute to the generation of a diagnostic score for the rapid and accurate identification of candidates for rapid molecular screening. However, MDS patients with both, *SF3B1* mutations and del(5q), exhibit immunophenotypic features more consistent with isolated del(5q) MDS, with decreased numbers of erythroid progenitors, higher CD71 expression and lower CD71 CV, suggesting that these patients may gain benefit from lenalidomide, rather than from luspatercept as first-line treatment (168).

Moreover, coexistence of monosomy 7 and *TP53* mutation are mostly related to increased CD14 expression on maturing granulocytic cells and to aberrant expression of T-cell antigens within the myeloid lineage, respectively (167, 172). These adverse genomic abnormalities, even when present alone, are usually enough to categorize patients as high-risk and direct treatment decision towards the use of hypomethylating agents. Immunophenotypic monitoring of lower-risk MDS patients would enable the timely suspicion of disease progression and the initiation of the appropriate treatment.

On the other hand, immunophenotypic patterns can provide prognostic information for treatment response. Thus, patients with lower risk MDS, and ≥3% CD117 expressing erythroid precursors are strongly associated with a favorable response to ESA treatment, transfusion independence and longer progression free survival (PFS) (178). Moreover, it was recently shown that erythroblastic predominance without CD41/cyCD41−positive blasts predicts for a favorable prognosis in MDS/AML patients treated with AZA (179) and that emergence of CD41+ blasts, is often accompanied by disease and/or cytogenetic progression (108). Lastly, for both, AZA and lenalidomide treatment, response monitoring using an immunophenotypic approach has been shown to be feasible (153, 176) and improved immunophenotypic features after AZA were found to predict longer duration of hematological response (176). However, these should be further substantiated and confirmed in prospective clinical studies.

Overall, even though several scoring systems for diagnosis and/or prognosis encompassing both, genomic and immunophenotypic data, the latter have not yet implemented and standardized, but steps are being taken to extract valuable information, that MFC can offer and translate our combined knowledge to clinical benefit for our patients.

## Challenges and opportunities for incorporating novel technologies into MDS research and clinical practice

The journey from CHIP to pre-leukemic syndromes, such as MDS or MPN, and eventually AML, is driven by the progressive selection of mutated clones, a process known as clonal evolution. Conventional “bulk” sequencing is limited by its ability to only analyze entire- cells populations, inevitably falling short for research in MDS, a disease with heterogenous cell populations and rare blast cells in certain cases. Single-cell technologies appear as ideal tools, both, to investigate the highly connected and plastic immune system, as well as to overcome the above problems, arising from clonality and heterogenicity in MDS (180). The high resolution of single-cell technology allows the study of even small cell groups with shared features, while high statistical power is provided by the large throughput of some sequencing platforms (181, 182).

As far as proteomics are concerned, recent sequencing-based techniques simultaneously quantify cell surface protein and transcriptomic data within a single cell readout (183, 184), thus overcoming the RNA-seq error in predicting protein expression. Moreover, mass spectrometry, using antibodies labeled with heavy metals detected by a mass spectrometer, has the advantage of simultaneous detection of around 40 parameters in each cell for millions of cells (180). This technique is considered suitable for tumor immune microenvironment research, since it can characterize subpopulations of immune cells with previously unknown alterations, and it can offer unbiased identification of HLA-neoantigens (185). Behbehani et al. attempted to depict the profile of MDS with mass spectrometry and they reported that it enabled the detection of aberrant surface markers at high resolution, detecting aberrancies in 27/31 surface markers, encompassing almost every previously reported MDS surface marker aberrancy (186).

Epigenomics have also recently adapted to single-cell applications, enabling the elucidation of DNA-methylation patterns, chromatin regions available for transcription factors activity, chromosomal conformation and histone modifications/binding sites in even thousands of individual cells. However, these methods are limited to cover specific regulatory regions (180).

On the other hand, MFC is a well-established technique but has not yet reached its full potential in MDS. AI, machine learning algorithms and other automated clustering techniques, trained from well-defined patient data, are being applied to large datasets enhancing diagnostic accuracy, providing repeatability, objectivity and a high sensitivity and specificity in detecting MDS (107, 187) or even highlight the predictive value of the detection of dysplasia for the response to treatment in AML cases (188) and could prove a new asset in everyday clinical practice in the future.

## Limitations and unresolved issues

Even though excessive studies in the field show promising results for the broadened application of MFC scores in routine clinical practice, the standardization of instruments and panels is far from being universally achieved and thus MFC in MDS is still a field of investigation for the implementation of suggested strategies and algorithms in routine clinical practice for the bigger part of the world. Either due to financial reasons or the questioning of the value of extensive MFC panels and time consuming analyses or the lack of expertise in some cases MFC is still heterogeneously used in the diagnostic process or follow up of MDS and suspected MDS cases, acknowledged only as a complementary tool for specific cases (177). Furthermore, the luxury of time in general when investigating suspected MDS; in contrast to AML where MFC is an established diagnostic method mainly for the rapid results, allows for the waiting of the molecular or cytogenetic results to confirm the clonal hematopoiesis. The heterogeneity of the disease, along with the similarities it exhibits with other clinical entities resulting in faulty hematopoiesis, is a co-founding factor for clinicians to rely on more definite tests (such as molecular or cytogenetic alterations) rather than immunophenotypic patterns indicative of dysplasia. Davydova et al (189) has reported that deviations of MFC parameters found in the control group can decrease the specificity of MFC scores when detecting dysplasia. Patients with PNH had increased levels of CD34^+^CD7^+^ myeloid cells. Aplastic anemia and PNH were characterized by a high proportion of CD56^+^ cells among CD34^+^ precursors and neutrophils. A second problem is that the proportion of MDS-related features increased with the progression of MDS, meaning that flow cytometry can detect more dysplastic immunophenotypic aberrancies when the disease is generally easier to detect. The highest number of CD34^+^ blasts was found in MDS with excess blasts. On the contrary, in 39 low-grade MDS – in the category where the most borderline cases belong, the sensitivities were 53.8%, 61.5%, and 71.8% for Ogata score, Wells score, and iFS, respectively, not addressing adequately the diagnostic problem that clinicians face when they investigate a suspected MDS case with rare blast cells.

Currently, both, genomic profiling and MFC are in the quiver of the physician for both, diagnosis and prognosis, but since NGS is still a high-cost technique and low-income countries are struggling to adapt (190), the association of specific phenotypic patterns with driver mutations in MDS, could serve as an indication to guide the selection of patients for mutational screening, in an effort to achieve optimal risk stratification.

## Conclusions and perspectives

MFC is a standard of care in the screening of patients with cytopenia, with or without dysplasia. For more than a decade, recommendations from the European LeukemiaNet have incorporated MFC as an additional tool in the diagnostic workup of MDS, compensating for the lack of specificity and repeatability in the morphological evaluation of dysplastic features (191). Bacher et al. showed that clonal chromosomal aberrations were detected in 14.3% of patients without suspicion of MDS by cytomorphologic evaluation during diagnostic work up, demonstrating the need of MFC to exclude MDS when the diagnosis is not confirmed by cytomorphological criteria (192). Various MFC scores have demonstrated up to 80% accuracy in discriminating between MDS and other nonclonal conditions with dysplastic morphological features (146). Further studies have highlighted the association of specific immunophenotypic patterns with cytogenetic or molecular findings, suggesting the ability of MFC to raise suspicion for the existence of specific genetic markers thus guiding further testing and accelerating the establishment of the precise diagnosis perhaps in a more cost effective manner.

On the other hand, the recognition of the prognostic importance of specific immunophenotypic aberrancies, and their incorporation in a composite prognostic scoring system, including clinical, biological and genetic prognostic parameters might further increase the accuracy of patient’s prognostic categorization and treatment response prediction. The integration of prognostically adverse mutational profiles - detected with NGS or other genetic testing- and myelodysplastic features - assessed by MFC scoring- has been implemented in other neoplasms such as systemic mastocytosis and has redefined prognostically different patient groups (193). Although the genetic landscape of MDS is broader and thus, a similar approach is more difficult to be implemented, advances in both the molecular characterization of the syndromes and the technical limitations of MFC analysis might overcome current difficulties in the future. The two methodologies are rather complementary and enable the physician to elaborate the complex phenotype of the disease. To that end, various groups have reported that the distribution of VAFs of individually mutated genes did not correlate with blast percentages in cases of MDS and AML, suggesting that VAF and blast percentage are different metrics of leukemic burden and thus both contribute differently to accurate clinical evaluation (194, 195).

**Figure 2:**
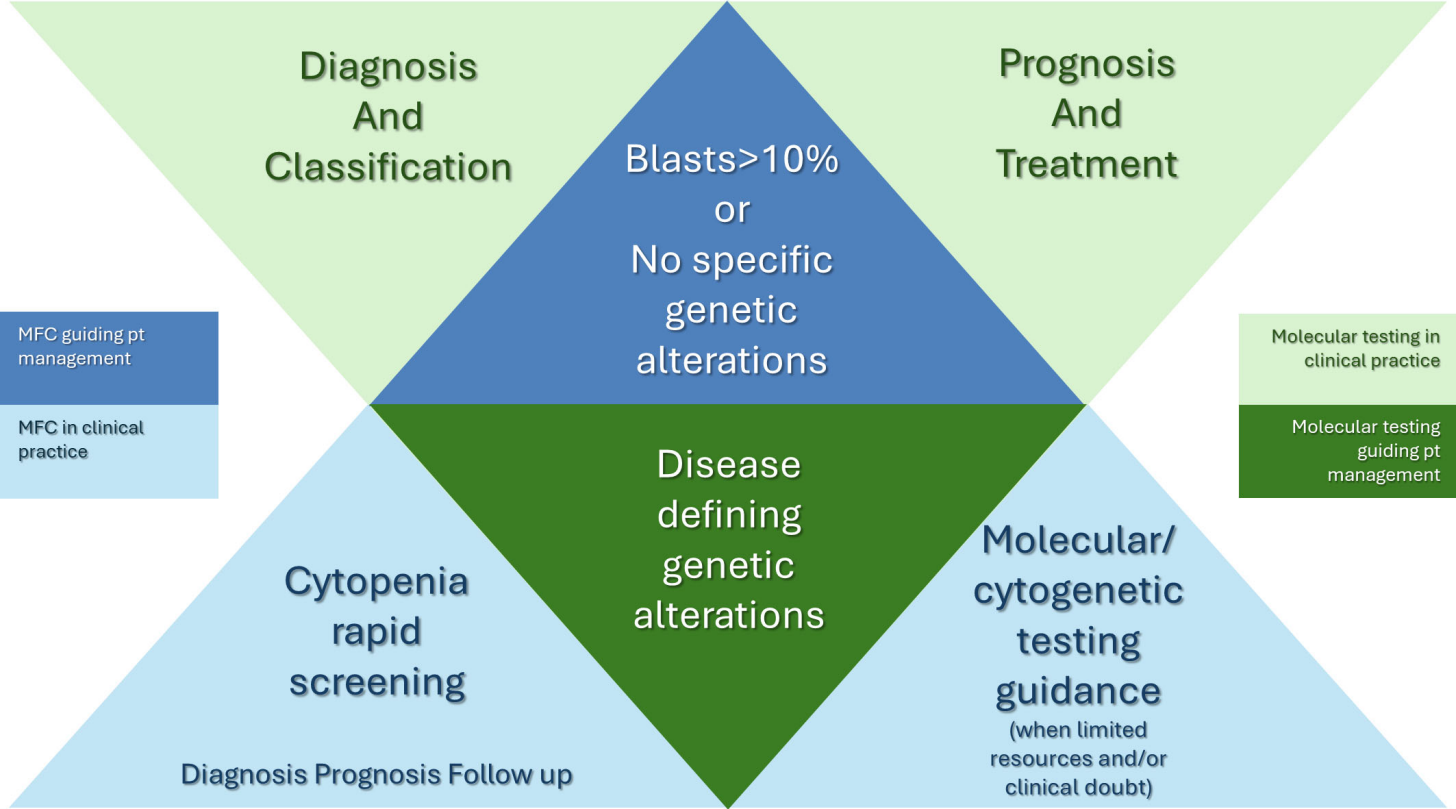
Schematic depiction of the interplay of MFC findings with cytogenetic and molecular testing in routine clinical practice. Clonal hematopoiesis is detected in more than 90% of MDS cases by cytogenetic or molecular alterations. These findings will establish the diagnosis but in terms of patient management, only certain disease-defining genetic alterations will constitute the basis of re-stratification of patients and individualize their treatment plan. On the other hand, MFC is currently used to investigate cytopenias and detect the cases in need for further cytogenetic or molecular testing. MFC can confirm or establish the diagnosis of MDS and guide clinical practice in cases with excess of blasts or patients with suspicion of MDS and no MDS-related genomic alterations.

Possibly, the establishment of new technologies and specifically of single cell – omics and a broadened implementation of machine learning algorithms for the analysis of merged data will bridge the gap between genotype and phenotype in the future and advance both, our understanding of the disease and our clinical practice. Until then, MDS poses a complex and delicate clinical challenge and MFC has proven a useful and “handy” tool for everyday screening of suspected MDS patients and an invaluable test for individual cases with no other pathognomonic findings to establish the diagnosis and guide further testing (**Figure 2**).

## Glossary

ABCB7: ATP Binding Cassette Subfamily B Member 7
AI: Artificial intelligence
ALL: Acute lymphoblastic leukemia
AML: Acute myelogenous leukemia
ASXL1: Additional sex combs-like 1
BCOR: BCL6 corepressor
BM: Bone marrow
CBL: Casitas B-lineage Lymphoma
CCUS: Clonal cytopenia of undetermined significance
CHIP: Clonal hematopoiesis of indeterminate potential
CLL: Chronic lymphocytic leukemia
CMML: Chronic myelomonocytic leukemia
CSNK1A1: Casein kinase 1 alpha 1
CV: Coefficient of variation
DDX41: DEAD-box RNA helicase-1 gene
DMI: Dysmyelopoiesis flow score
DNMT3A: DNA methyltransferase 3A
EIF2AK1: Eukaryotic translation initiation factor 2-alpha kinase 1
ELN: European LeukemiaNet
EPO: Erythropoietin
ESA: Erythropoietin stimulating agents
ETV6: ETS variant transcription factor 6
EuroFlow: EuroFlow Consortium
EZH2: Enhancer of zeste homolog 2
FAB: French-American-British
FCSS: Flow cytometry scoring system
FISH: Fluorescence *in situ* hybridization
FLAER: Fluorescein-labeled proaerolysin
Fli1: Friend leukemia virus integration 1
FlowSOM: Flow-Self Organizing Maps algorithm
FLT3: Fms-like tyrosine kinase 3
GATA1/2: GATA-binding factor ½
GPI: Glycosylphosphatidylinositol
GPIIb: Glycoprotein IIb
HLA: Human leukocyte antigens
HSCT: Hematopoietic stem cell transplantation
ICC: International Consensus Classification
ICUS: Idiopathic cytopenia of undetermined significance
IDH1/IDH2: Isocitrate dehydrogenase 1/2
IDUS: Idiopathic dysplasia of unknown significance
iFS: Integrated flow cytometric score
iMDS Flow: International MDS Flow Working Group
IPSS: International prognostic scoring system
IPSS-M: Molecular IPSS
IPSS-R: Revised IPSS
JAK2: Janus kinase 2
KDM6A: Lysine demethylase 6A
KRAS/NRAS: Kirsten/neuroblastoma rat sarcoma viral oncogene homolog
LAIP: Leukemia associated immunophenotype
LF: Lactoferrin
LFS: Leukemia free survival LFS
LOH: Loss of heterozygosity
MDS: Myelodysplastic syndromes/neoplasms
MDS/MPN: Myelodysplastic/myeloproliferative neoplasms
MDS-biTP53: MDS with biallelic TP53 inactivation
MDS-EB: MDS with excess blasts
MDS-SF3B1: MDS with low blasts and SF3B1 mutation
MFC: Multiparameter flow cytometry
MFI: Mean fluorescent intensity
MLL: Mixed-lineage leukemia
MPN: Myeloproliferative neoplasms
MPO: Myeloperoxidase
MRD: Measurable clonal residual disease
NGS: Next Generation Sequencing
NPM1: Nucleophosmin 1
OS: Overall survival
PCNA: Proliferating cell nuclear antigen
PFS: Progression free survival
PNH: Paroxysmal nocturnal hemoglobinuria
PPOX: Protoporphyrinogen oxidase
RBC: Red blood cells
RNA-seq: RNA sequencing
RPS14: Ribosomal Protein S14
RUNX1: Runt-related transcription factor 1
SF3B1: Splicing factor 3B unit 1
SRFS2: Serine and arginine-rich splicing factor 2
SSC: Side scatter
STAG2: Stromal antigen 2
TET2: Tet methylcytosine dioxygenase 2
Th17: T helper type 17 cells
Th22: T helper type 22 cells
TP53: Tumor protein p53
Tregs: Regulatory T cells
U2AF1: U2 small nuclear RNA auxiliary factor 1
UBA1: Ubiquitin like modifier activating enzyme 1
VAF: Variant allele frequency
WHO: World Health Organization
WPSS: WHO classification based prognostic scoring system
WT1: Wilms tumor 1
ZRSR2: Zinc finger CCCH-type, RNA binding motif and serine/arginine rich 2
